# SrCr_x_Fe_12-x_O_19_ nanoceramics as an effective catalyst for desulfurization of liquid fuels: Green sol-gel synthesis, characterization, magnetic and optical properties

**DOI:** 10.1371/journal.pone.0162891

**Published:** 2017-05-11

**Authors:** Samira Mandizadeh, Faezeh Soofivand, Samira Bagheri, Masoud Salavati-Niasari

**Affiliations:** 1 Institute of Nano Science and Nano Technology, University of Kashan, Kashan, I. R. Iran; 2 Nanotechnology & Catalysis Research Centre (NANOCAT), IPS Building, University of Malaya, Kuala Lumpur, Malaysia; Institute of Materials Science, GERMANY

## Abstract

In this work, SrCr_x_Fe_12-x_O_19_ (x = 0.0, 0.5, 1.0, 1.5) nanostructures were successfully synthesized by sol-gel auto-combustion method, and different aminoacids were used as green reductants. Various analysis results show that SrCr_x_Fe_12-x_O_19_ nanoparticles synthesized successfully.The present study shows that SrCr_x_Fe_12-x_O_19_ nanoparticle could be used as adsorbent for the desulfurization of liquid fuels. Increasing of nanoparticles concentration was caused to increase the adsorption rate of sulfur contents of fuel. The adsorption rate of sulfur contents of fuel in various concentrations 4.5, 9.5, and 18.5 g. L ^-1^ of SrCr_x_Fe_12-x_O_19_ nanoparticles in solution was estimated about 39, 50, and 62% for 30 min, respectively. The results of catalytic tests reveals that SrCr_x_Fe_12-x_O_19_ nanoparticles have the potential to be used as a new kind of semiconductor catalysts for the desulfurization of liquid fuels. Magnetic property of the final sample was measured at room temperature by a vibration sample magnetometer (VSM) and shown that the intrinsic coercivity of product is about 6000 Oe and it exhibits characteristics of single magnetic domains (M_r_/ M_s_ = 0.53).

## Introduction

Nanostructure materials are used in various fields such as photocatalytic, superconductors, nanoelectronicssuch as photocatalytic, superconductors, nanoelectronics and supercapacitors [1–3]. The properties of nanomaterials depend on their composition, morphology, size and size distribution. Among, it was found that SrFe_12_O_19_ has comprehensive applications in magnetic recordings, microwave devices and permanent magnets [4–6]. In recent years, numerous methods have been reported for the synthesis of SrFe_12_O_19_ nanostructures such as the hydrothermal [7, 8], sol-gel [9–11], reverse micelle [12] and co-precipitation [13, 14].

Recently, simple and inexpensive methods have been replaced by complex and expensive routes that can control the shape and size of nanostructures, also, due to the environmental worries, using of natural sources instead of chemical compounds has particular importance. Hence, we tried using amino acids as a natural source to use of green chemistry benefits. On the other hand, environmental concerns have driven the need to remove sulfur-containing compounds from light oil. One of main component in liquid fuel is sulfur compounds such as SO_2_ and H_2_S that are harmful for environmental and human healthy, so, much attention has been recently devoted to this process. In present work, removal of sulfur compounds in liquid fuels using SrCr_x_Fe_12-x_O_19_ nanoparticles as an effective catalyst was reported. The obtained results showed that the mechanism of desulfurization follows Freundlich isotherm that is another form of the Langmuir approach for adsorption on a heterogeneous surface. Furthermore, the sol-gel route has used as a versatile technique for preparing chemical compounds and inorganic materials that possess advantages such as: chemical homogeneity, easy component adjustment, low calcination temperature and low cost, in addition to sol-gel route is one of methods for synthesis of nanomaterials [15, 16].

The SrFe_12_O_19_ with high crystalline and small particle size is synthesized by sol-gel method [17, 18]. The structure of M-type hexagonal is stacked alternatively by spinel (S = Fe_6_O_8_^2+^) and hexagonal (R = MFe_6_O_11_^2-^) layers. The O ^2-^ exist as close-packed layers, with the M ^2+^ substituting for an O^2-^ ion in the hexagonal layer. The Fe^3+^ are distributed in the five interstitial crystallographic sites of the close-packed layers, i.e. three octahedral (2a, 12k and 4f_2_), one tetrahedral (4f_1_), and one trigonal bipyramid (2b). The three parallel (2a, 12k and 2b) and two antiparallel (4f_1_ and 4f_2_) sub-lattices, which are coupled with super-exchange interactions through the O^-2^ ions, form the ferrimagnetic structure [19–21]. In many works, various ions are replaced Fe^3+^ ions [22–24]; this substitution is carried out in order to improvement of properties.

Herein, SrCr_x_Fe_12-x_O_19_ (x = 0.0, 0.5, 1.0, 1.5) nanostructures were synthesized by using various amino acids as fuel, reducing and capping agent via sol-gel auto-combustion technique. In addition, the effect of various parameters such as type of amino acid and chromium ions concentration that is substituted by Fe^+3^ cations on the particle size, morphology and purity of product was studied. The as-produced nanostructures were characterized by scanning electron microscopy (SEM), Fourier transform infrared spectroscopy (FTIR), energy dispersive X-ray spectroscopy (EDS) analysis and powder X-ray diffraction (XRD). Magnetic property of the final sample was measured at room temperature by a vibration sample magnetometer (VSM) and shown that the intrinsic coercivity of product is about 6000 Oe. The results of catalytic tests reveals that SrCr_x_Fe_12-x_O_19_ nanoparticles have the potential to be used as a new kind of semiconductor catalysts for the desulfurization of liquid fuels.

## Materials and methods

### Characterization

All the chemicals used in this work were commercially available and employed without further purification. FT-IR spectra were recorded on Magna-IR, spectrometer 550 Nicolet in KBr pellets in the range of 400–4000 cm^–1^. Powder X-ray diffraction (XRD) patterns were collected from a diffractometer of the Philips Company with X’PertPro monochromatized Cu Kα radiation (λ = 1.54 Å). Microscopic morphology of the products was studied by FESEM (Mira3 tescan) and TEM (HT-7700). The energy dispersive spectrometry (EDS) analysis was studied by XL30, Philips microscope. The magnetic properties of the samples were detected at room temperature using a vibrating sample magnetometer (VSM, Meghnatis Kavir Kashan Co., Kashan, Iran).

### Preparation of nanostructures

Sr(NO_3_)_2_, Fe(NO_3_)_3_. 9 H_2_O, Cr(NO_3_)_3_. 9 H_2_O and various amino acids were used as starting materials. SrCr_x_Fe_12-x_O_19_ (x = 0.0, 0.5, 1, 1.5) nanostructures were synthesized through the reaction between Sr(NO_3_)_2_, Fe(NO_3_)_3_. 9 H_2_O, Cr(NO_3_)_3_. 9 H_2_O with molar ratio of 1: 12: x (x = 0.5, 1 and 1.5), respectively. At first, 0.2 g (1 mmol) Sr(NO_3_)_2_ was dissolved in 75 ml of distilled water, and then aqueous solutions including stoichiometric amount of Fe(NO_3_)_3_. 9 H_2_O (4.5 g) and Cr(NO_3_)_3_. 9 H_2_O was added to it. The resulted solution was vigorously stirred at 50°C for 30 min. Then, the appropriate amount from amino acid (glutamine, cysteine, valine and leucine) was dissolved in a minimum amount of de-ionized water (the molar ratio of amino acid to Sr(NO_3_)_2_ was fixed at 13). Finally, thermal dehydration was done and the obtained powder disinterred at 800, 900 and 1000 ^O^C for 120 min. The as-prepared products were characterized by XRD, SEM, EDS, FT-IR and TEM.

### Preparation of sample for catalytic process

Sulfur content of fuel has been decrease to low levels by environmental regulation in worldwide with the aim of improving air quality. In this research, SrCr_x_Fe_12-x_O_19_ nanoparticles (sample no. 6) were used as new adsorbent. The present study shows that SrCr_x_Fe_12-x_O_19_ nanoparticles could be used as adsorbent for the desulfurization of liquid fuels. Total sulfur content was measured using petrotest calorimetric bomb C5000.according to ASTM D-1266. Batch experiment for determination of the kinetic models are used. Kinetics of sulfur on SrCr_x_Fe_12-x_O_19_ nanoparticles carried out at 35 ^O^C. Different concentration of SrCr_x_Fe_12-x_O_19_ nanoparticles were brought in contact with 30 ml sample at 35 ^O^C.

## Results and discussion

To investigate the effect of different parameters on the morphology, particle size and purity of the products, the various experiments were performed. All of the preparation conditions were illustrated in Table 1.

**Table 1:** Preparation conditions of samples 1–8.

Sample no.	Kind of Amino acids	Amount of Cr^+3^ / Sr^+2^	Morphology and Particle size
1	-	0	Irregular shapes; 300 nm
2	Glutamine	0	Irregular shapes; 50 nm- 500 nm
3	Cysteine	0	Hexagonal shapes; 200–400 nm
4	Valine	0	Hexagonal and uniform shapes; 150 nm
5	Leucine	0	Irregular shapes and particle; 50 nm- 500 nm
6	Valine	0.5	Uniform particle; 50 nm-
7	Valine	1	Irregular shapes; 200 nm
8	Valine	1.5	Irregular large shapes and fine particles

### SEM images

**Fig 1:**
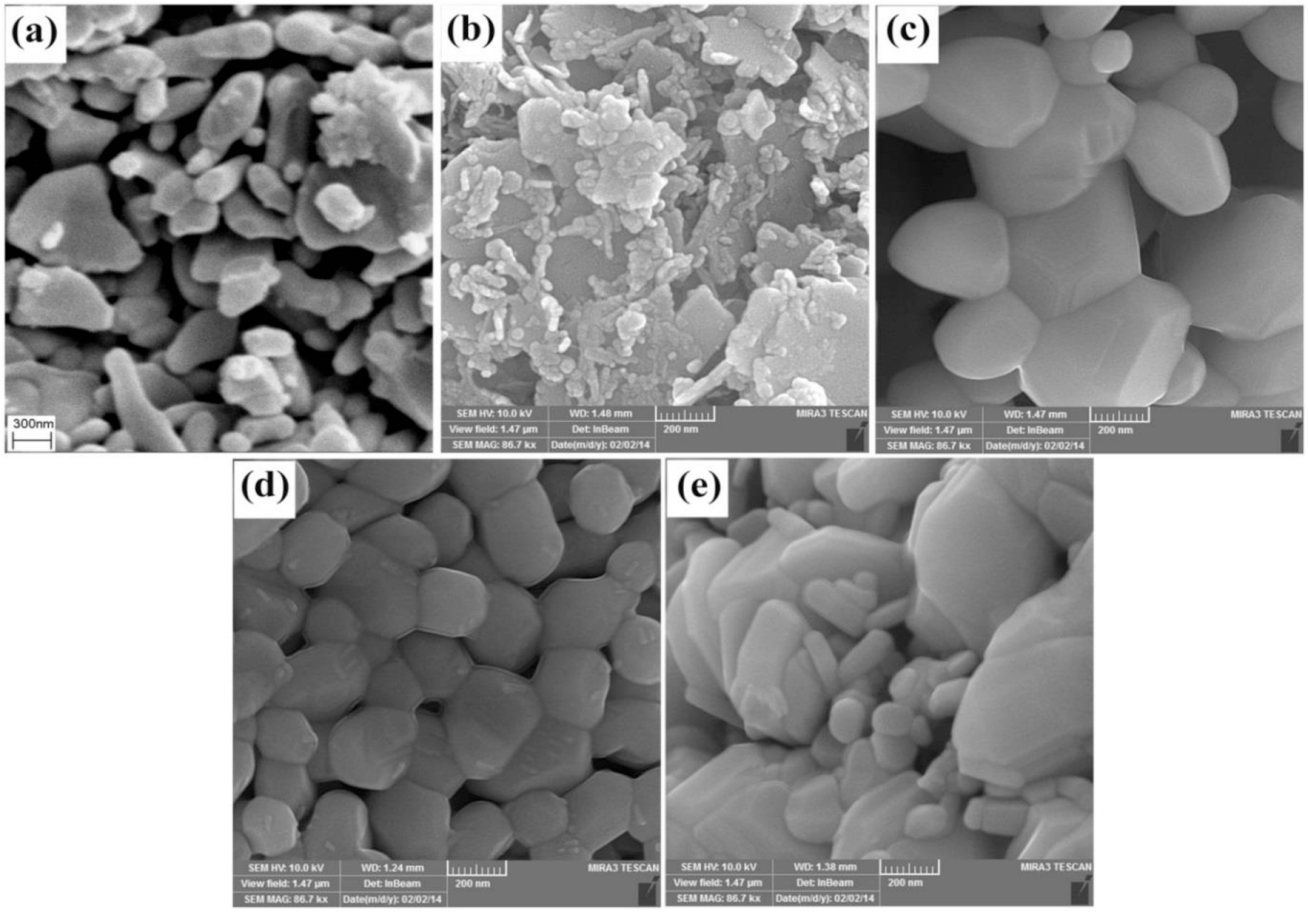
SEM image. (a) sample 1, (b) sample 2, (c) sample 3, (d) sample 4 and (e) sample 5.

The morphology of the products (SrFe_12_O_19_) prepared by various aminoacids is examined by SEM (Fig 1). The sample prepared without aminoacid was considered as blank sample (sample no. 1), SEM image related to this sample shows the agglomerated nanostructures (Fig 1A). SEM images of the samples prepared with various amino acids such as glutamine (sample no. 2), cysteine (sample no. 3), valine (sample no. 4) and leucine (sample no. 5) are shown in Fig 1B–1E, respectively. In Fig 1A–1C and 1E), the obtained structures are irregular and hetrogenous with different particle size. Fig 1D depicts the hexagonal and uniform structures with particle size about 100–200 nm. The valine is a branched-chain amino acid that creates more steric hindrance than the other used amino acids in this process, so, was chosen as a desired reducing agent.

**Fig 2:**
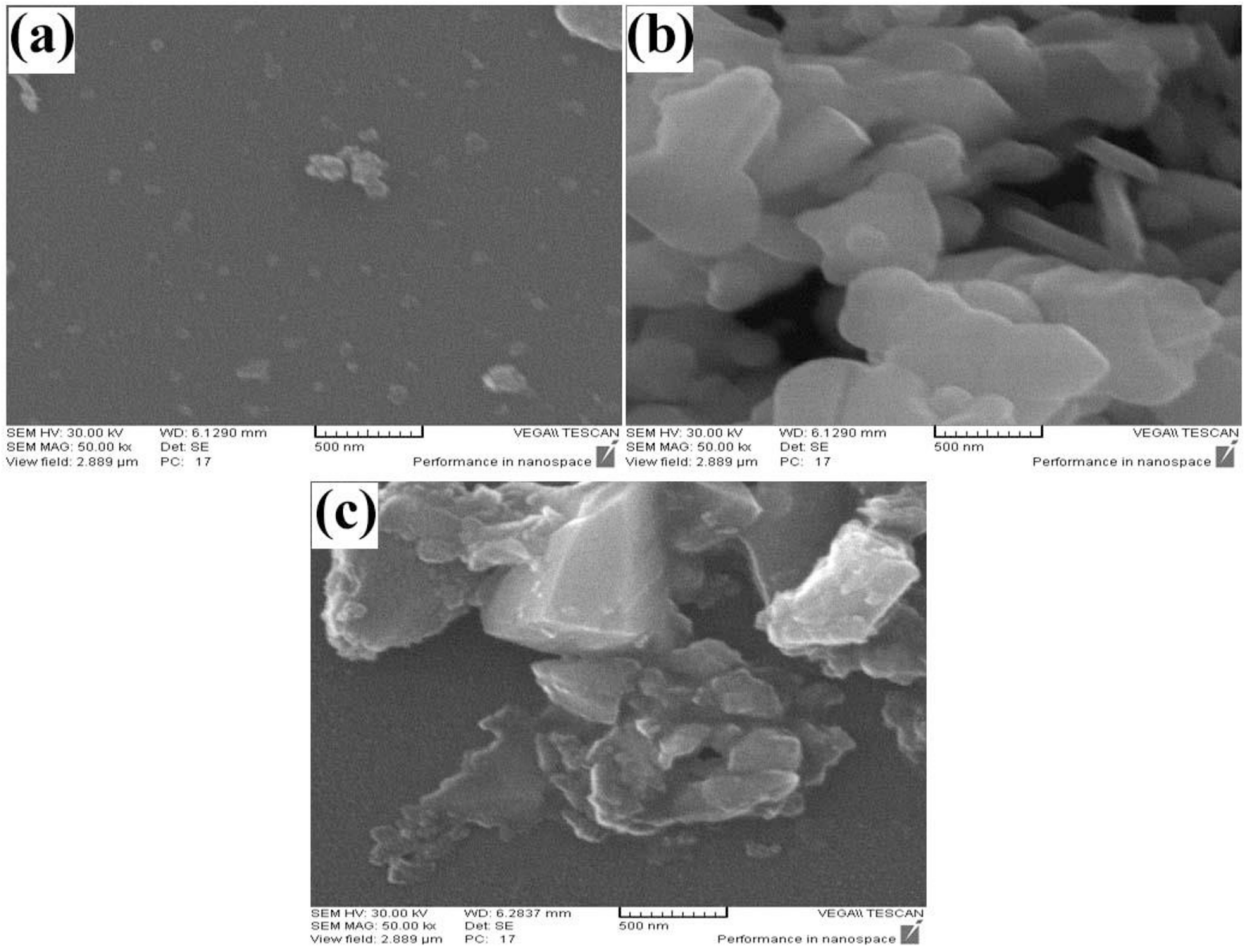
SEM image. (a) sample 6, (b) 7, and (c) 8.

The morphology of the SrCr_x_Fe_12-x_O_19_ (x = 0.5, 1.0, 1.5) micro/nanostructures is examined by SEM (Fig 2). As shown in this figure, fine and uniform particles were formed for sample 6 (Fig 2A). Morphology of sample 7 is sheet-like structures that have micrometer scale (Fig 2B) and the irregular structures can be shown in Fig 2C that related to sample 8. By considering these SEM images, sample 6 is considered as optimum sample and morphology, because the fine and homogenous particles were formed that their size is about 40 nm (Fig 2A). But purity of products is so important and XRD patterns of samples must be investigated.

### X-ray diffraction

**Fig 3:**
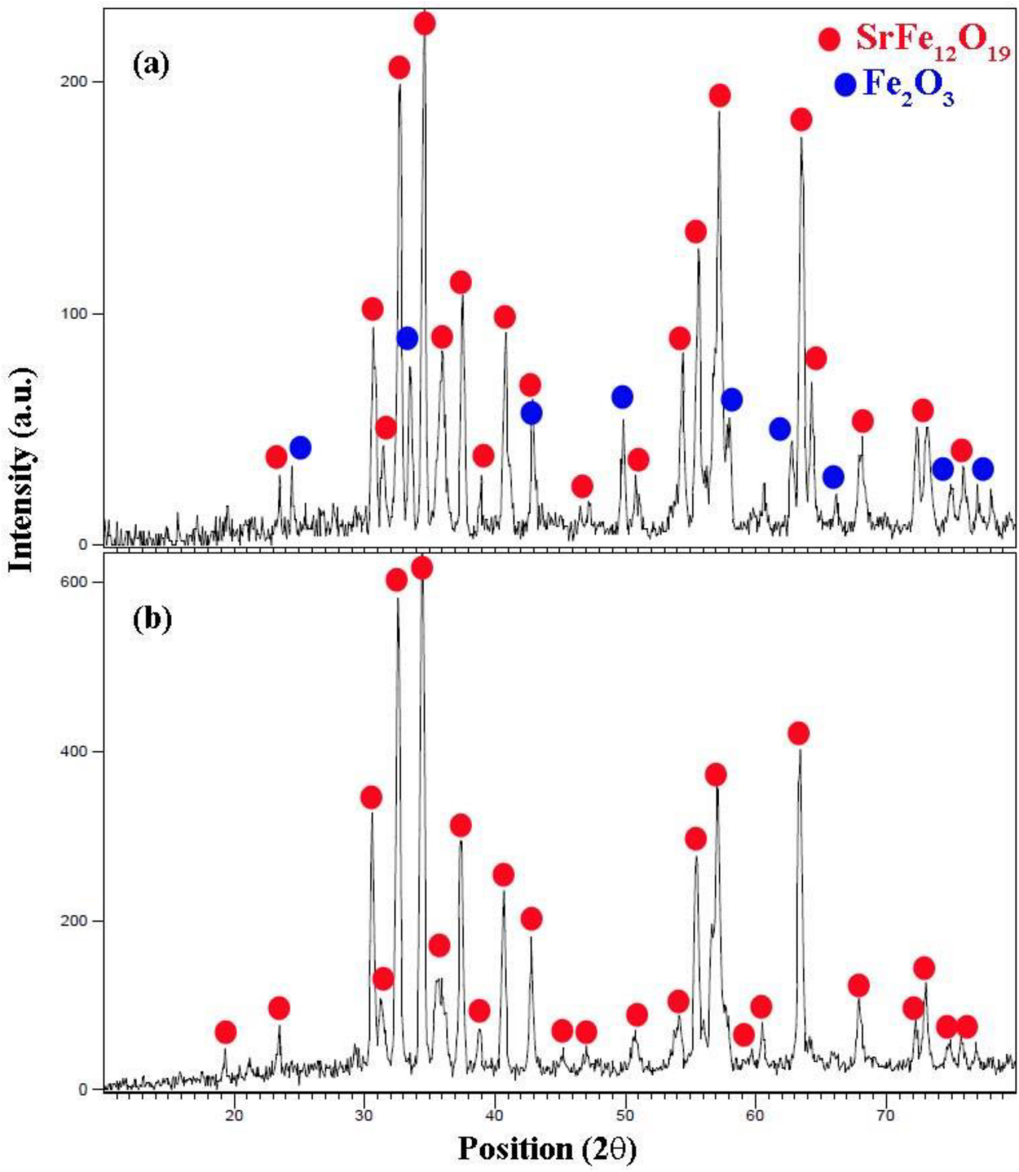
XRD pattern. (a) sample 4, and (b) sample 6.

**Fig 4:**
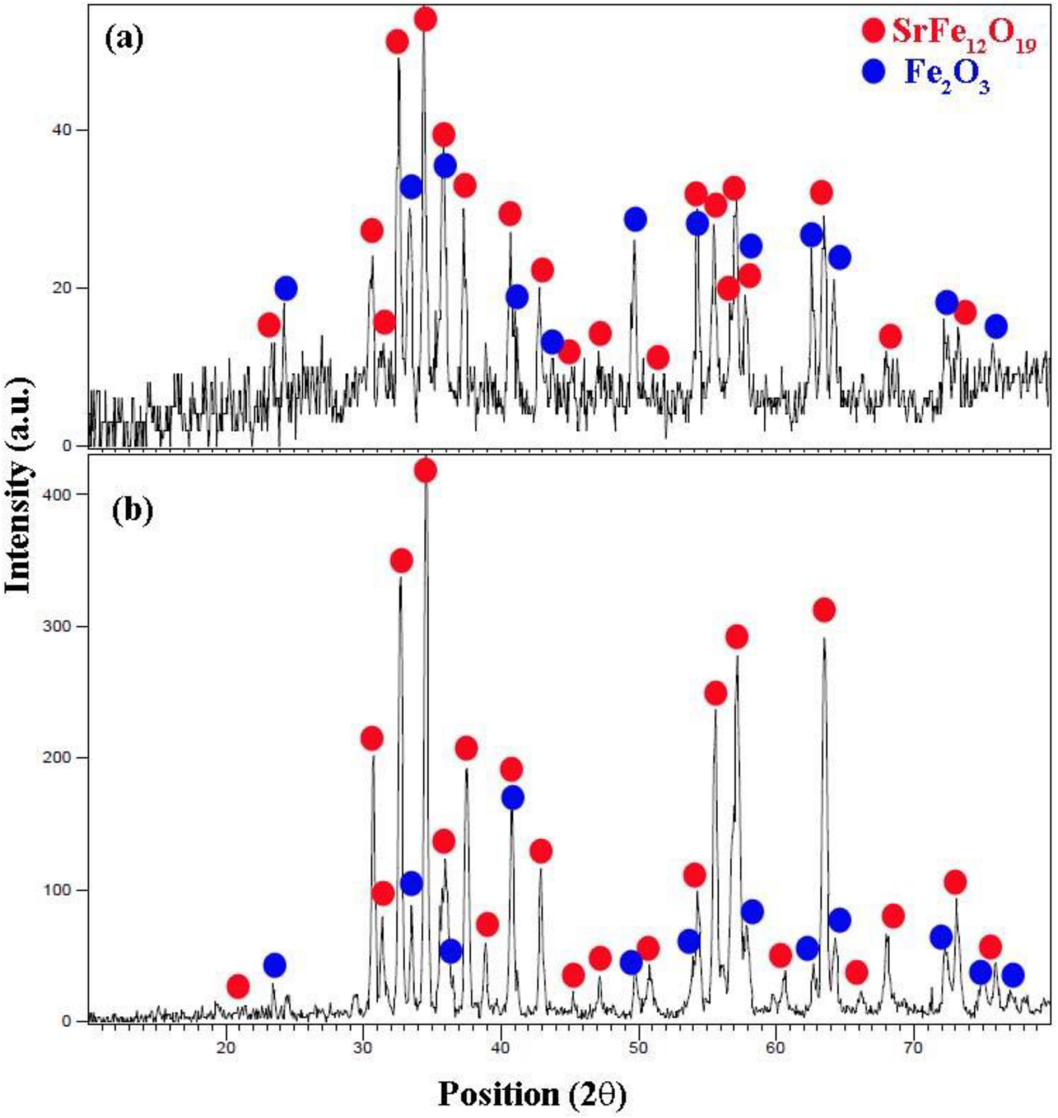
XRD pattern. (a) sample 7, and (b) sample 8.

The crystal structure of the as-prepared micro/ nanostructure is determined by XRD. Fig 3 shows XRD patterns of SrCr_x_Fe_12-x_O_19_ nanostructures synthesized by the reaction between Fe(NO_3_)_3_. 9 H_2_O, Sr(NO_3_)_2_ and valine in presence of various amounts of Cr(NO_3_)_3_ (Cr^+3^ / Sr^+2^ molar ratio = 0.0 (sample no. 4), 0.5 (sample no. 6), 1.0 (sample no. 7), 1.5 (sample no. 8)). The diffraction peaks of the products in Fig 3A, Fig 4A, and 4B can be indexed to rhomb-centered hexagonal Fe_2_O_3_ (JCPDS card 84–0306) that is shown with blue stars, and hexagonal SrFe_12_O_19_ (JCPDS 33–1340). The formation of Fe_2_O_3_ when the concentration of Cr^3+^ is ≥ 0.5 has also been reported by Fang et al. [24]. The formation of Fe_2_O_3_ can be explained on the basis of the Sr(NO_3_)_2_ poor solubility in water as compared to Fe(NO_3_)_3_ and its weaker coordination ability of reducing agents to Sr^2+^ ions as compared to Fe ^3+^ ions [25]. The diffraction peaks in the XRD pattern of sample 6 can be ascribed to hexagonal SrFe_12_O_19_ (JCPDS 33–1340) and remarkable diffractions of other compounds cannot be found in Fig 3B, this XRD pattern is related to sample that the molar ratio of Cr^+3^/ Sr^+2^ was 0.5. The size of crystallites is estimated from the Debye-Scherrer equation is about 45 nm.

### TEM image

**Fig 5:**
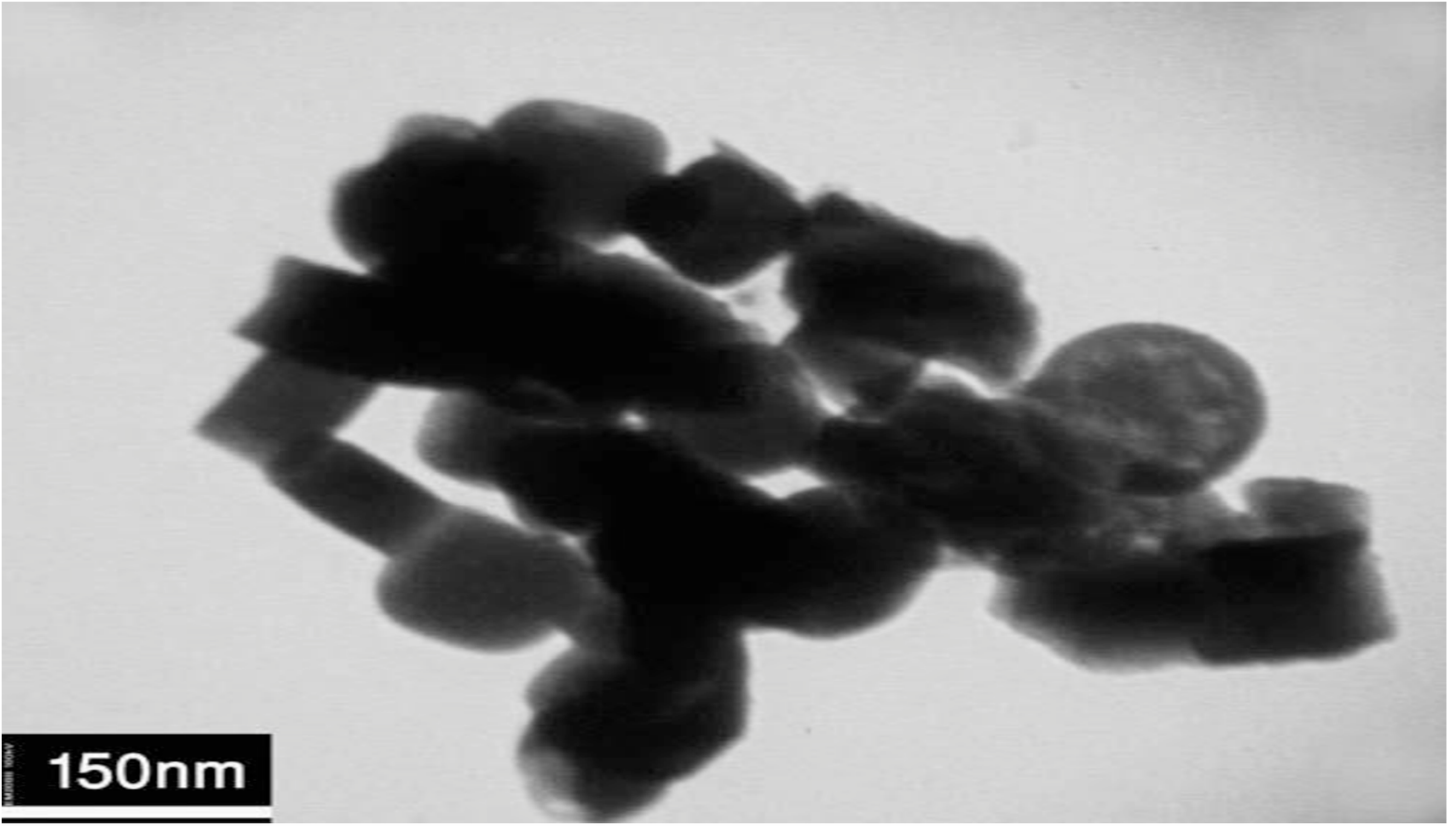
TEM image of sample 6.

Fig 5 shows a typical TEM image of sample no. 6. As shown in Fig 5 diameters of nanostructures are about 60 nm.

### EDS analysis and Fourier transform infrared spectroscopy

**Fig 6:**
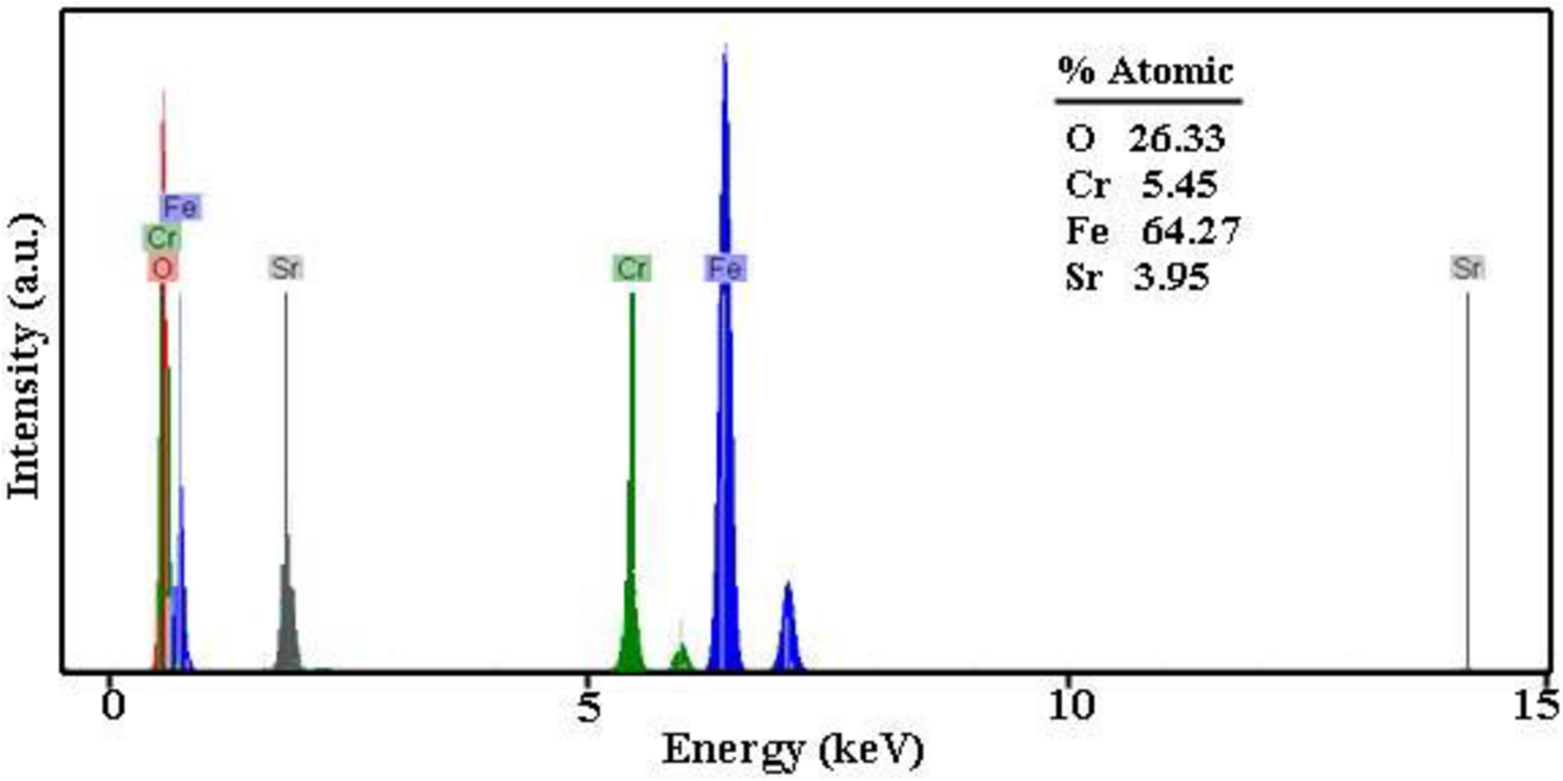
EDS spectrum of sample 6.

In the EDS spectrum of SrCr_x_Fe_12-x_O_19_ obtained from sample 6 (Fig 6), Sr (3.95%), Fe (64.27%), Cr (5.45%) and O (26.33%) elements are detected (Fig 6). There were no peaks for other impurities and the EDS results confirmed high purity of the synthesized SrCr_x_Fe_12-x_O_19_ nanostructures through this route.

**Fig 7:**
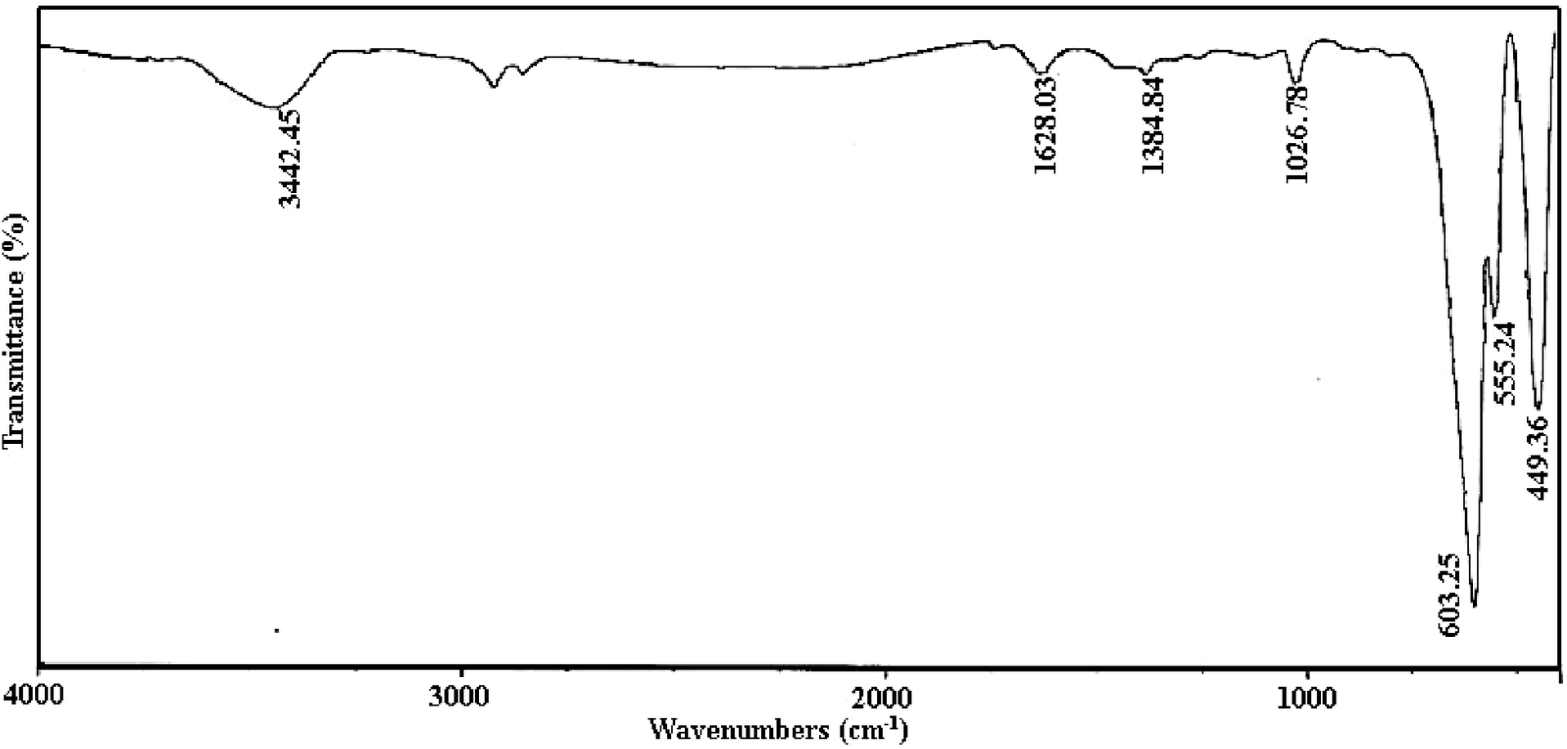
FT-IR spectrum of sample 6.

Fig 7 shows the FT-IR spectrum of the sample no. 6. The absorption characteristic peaks of SrCr_x_Fe_12-x_O_19_ (x = 0.0) were at 449.36 cm^−1^, 555.24 cm^−1^, and 603.25 cm^−1^ [26] and this spectrum demonstrated that the added Cr^3+^ didn’t change the intrinsic structure of SrFe_12_O_19_. However, the free O–H stretch vibration of the carboxyl group around 3444 cm^−1^. Furthermore, presence of the O–H group can be confirmed by peak shown at about 1026 cm^-1^ that related to in-plane-bend vibration and usually appears for carboxylic OH group that is bonded as non-hydrogen. Similarly, the absorption peak around 1620 cm^-1^ is assigned to the stretch vibrations of C = O group in amino acids (in this sample, the used amino acid was valine); however, when this group contribute to formation of hydrogen bonds, the related peak is transferred to higher wavelengths with lower energies [27]. By considering this result, can be said that the combustion reaction using of amino acid is completed and don’t occur any by-reactions. The band C–H/N–H bending vibrations are shown at about 1380 cm^-1^.

### XPS studies

**Fig 8:**
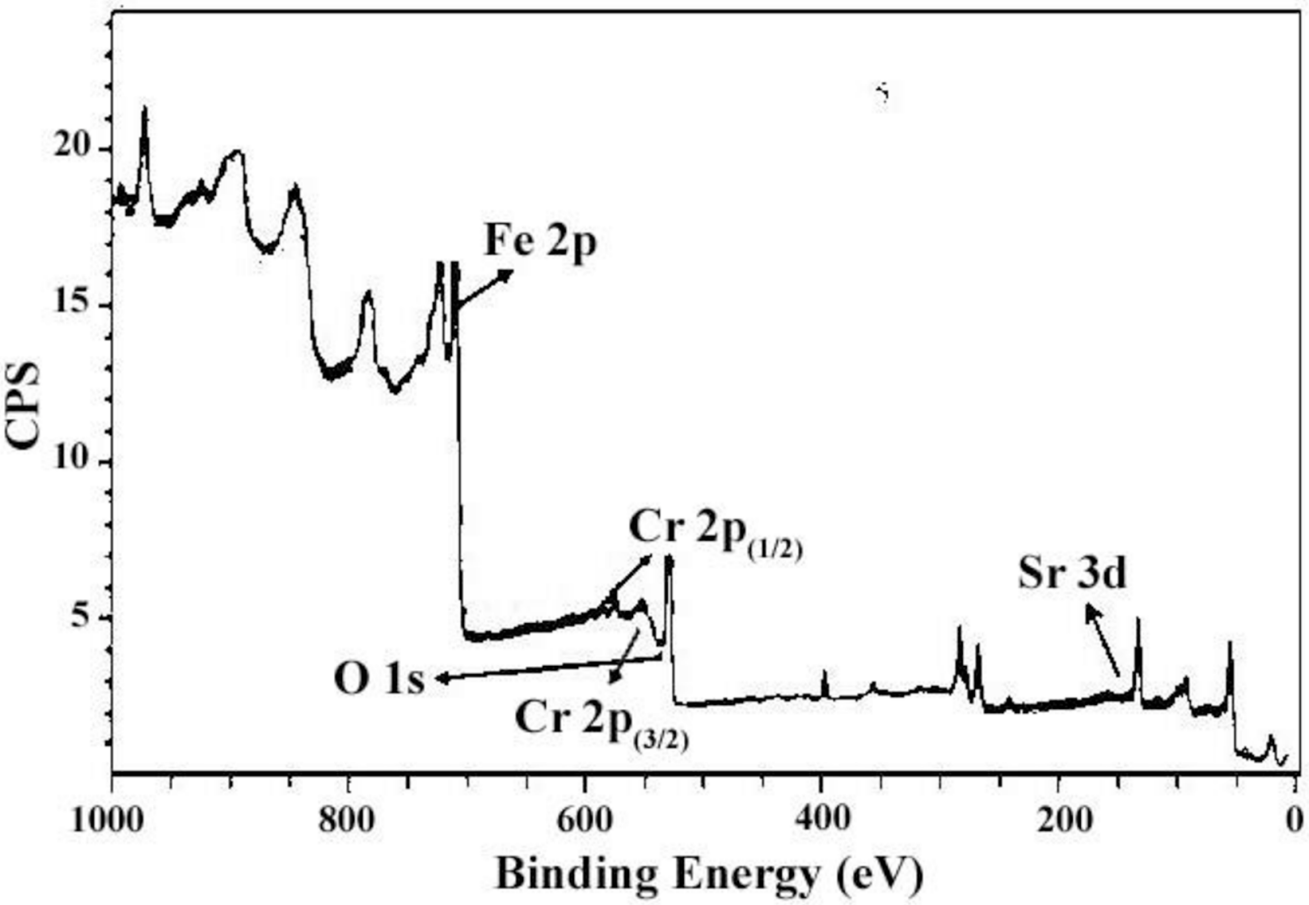
XPS spectrum of sample 6.

Fig 8 shows the XPS of sample no. 6. The formation of the obtained SrCr_x_Fe_12-x_O_19_ and presence of Cr^+3^ in this compound can be confirmed by this spectrum. The XPS spectrum indicates that the elements Sr, Fe, Cr and O were present in the SrCr_x_Fe_12-x_O_19_. The XPS of the Sr 3d, Fe 2p, Cr 2p, and O 1s levels were present in the following forms: (1) Sr 3d region (129–137 eV); (2) Fe 2p region (708–732 eV); and Cr 2p 1s region (570–581 eV) [28]. In this figure, the peak centered at 531.4 eV was attributed to the O 1s. The peaks located at about 134.2, 712, 574.5 and 580.2 eV were attributable to the Sr 3d, Fe 2p, Cr 2p(3/2) and Cr 2p(1/2), respectively. The peak centered at 531.4 is in accordance with the other reports for O 1s peak of the oxide-metal [29].

### UV-vis spectroscopy and band-gap studies

**Fig 9:**
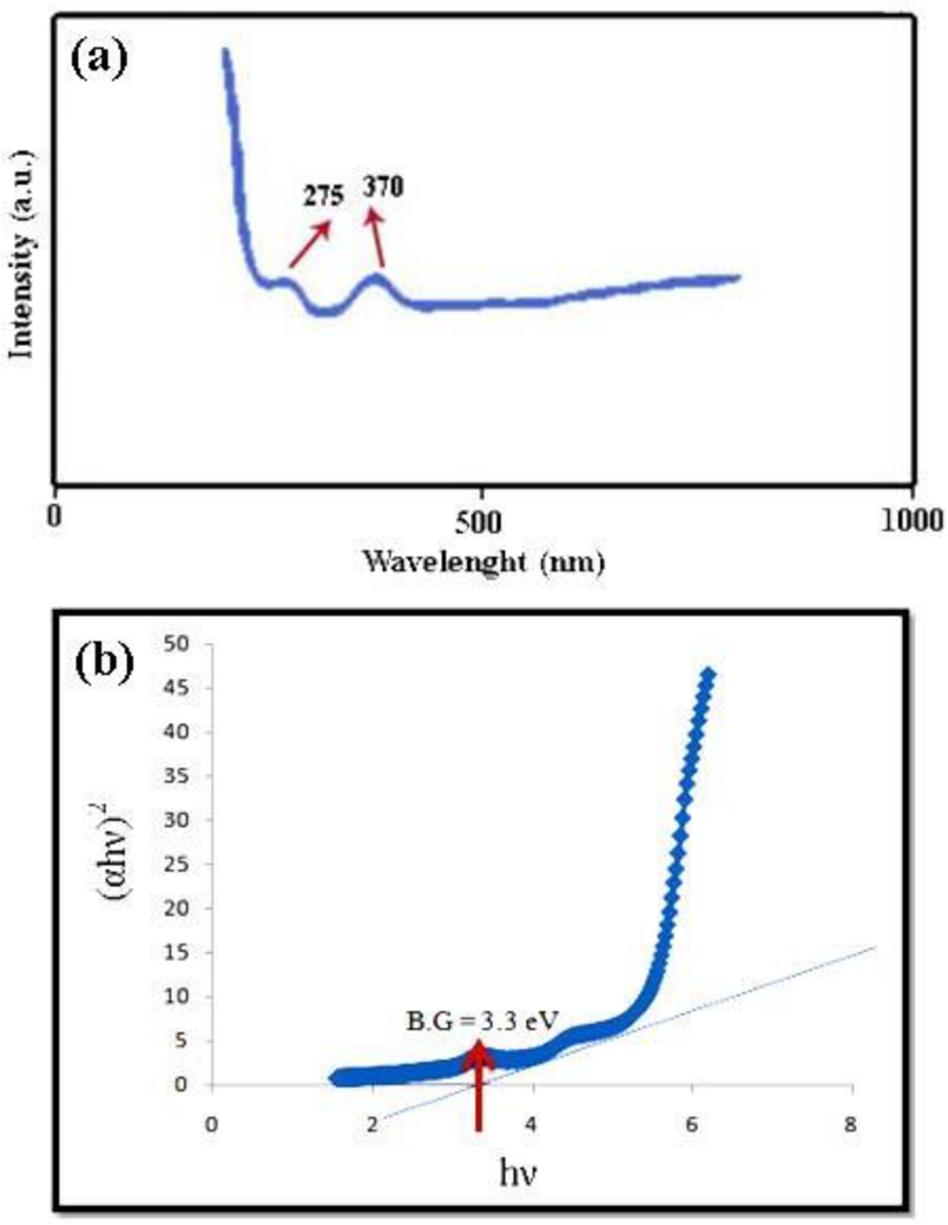
Adsorption studies. (a) UV–vis spectrum, and (b) curve (Ahυ) ^n^ versus hυ of sample 6.

Fig 9A shows the UV–vis absorption spectrum of the as-synthesized SrCr_x_Fe_12-x_O_19_ (sample no. 6). In this spectrum, two absorption peaks appeared around 275 and 370 nm, which is alike to Xie’s results [30]. Previous literature [31] reported that visible light sensitivity in these systems is due to Fe cations. The Fe^3+^ ion possessed a 3d^5^ configuration which had a sextet state in the octahedral crystal field. Meanwhile, very weak crystal field transitions were expected as these transitions were spinning, symmetry and parity forbidden [31, 32]. In addition, charge transfer transition from O^2−^ to Fe^3+^ normally gave rise to a strong absorption around 275 nm (see Fig 9A). Haart and his coworkers reported that absorption in the visible area is due to metal to metal charge transfer transitions (2Fe^3+^ → Fe^2+^+ Fe^4+^) [32].

Optical band gap (Eg) may be evaluated based on the optical absorption spectrum using the following equation [33]: (Ahυ)^n^ = B(hυ-Eg); Where hυ is the photon energy, A is absorbent, B is a material constant and n is 2 or 1/2 for direct and indirect transitions, respectively. The optical band gap for the absorption peak is obtained by extrapolating the linear portion of the (Ahυ) ^n^ curve versus hυ to zero (Fig 9B). No linear relation was found for n = 1/2, suggesting that the as-prepared SrFe_12_O_19_ are semiconductors with direct transition at this energy. The energy gap of sample no. 6 was measured by the data of UV-vis spectrum and reported about 3.3 eV. This value of energy gap confirms that SrCr_x_Fe_12-x_O_19_ nanostructures can be applied as an effective photocatalyst.

### Magnetic properties

**Fig 10:**
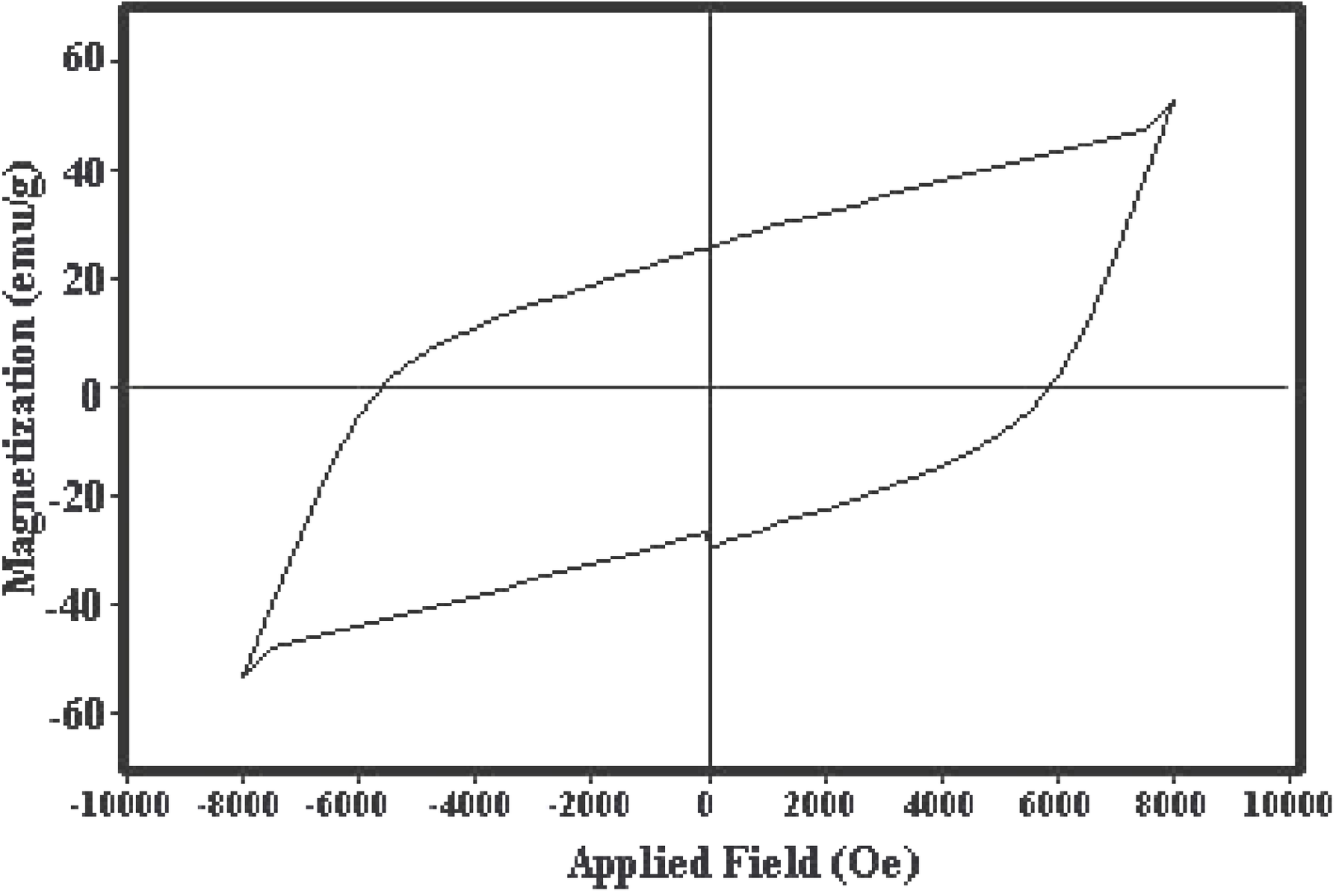
Magnetization versus applied magnetic field at room temperature for sample 6.

The magnetic properties of materials were investigated by VSM. The magnetic hysteresis loop was depicted in Fig 10. Magnetic hysteresis loop at room temperature has been recorded for sample no. 6. As shown, when chromium is doped to the Fe ^3+^-containing compounds, the saturation magnetization, M_s_ is decreased [34, 35] because the ion moment of Cr^3+^ (3μB) is lower than the ionized moment of Fe ^3+^ (5μB), so the exchange interactions are weaken. It is known that Cr^3+^ ions penetrate to octahedral sites-12k (up), 2a (up) and 4f_2_ (down), on the other hand, reduction of saturation magnetization with doping Cr ^3+^ indicates that the Cr^3+^ ions must be occupying 12 k and 2a sites in preference to 4f_2_ sites [36]. Also, when the Cr^3+^ is doping, coercivity increases due to increase in anisotropy field, which in turn increases the domain wall energy [37, 38]. On the other hand, the coercivity will increase with the increase of the morphology anisotropy. The intrinsic coercivity of sample no. 6 is about 6000 Oe and it exhibits characteristics of single magnetic domains $\left(\frac{Mr}{Ms}=0.53\right)$ [39].

### Catalytic activity

**Fig 11:**
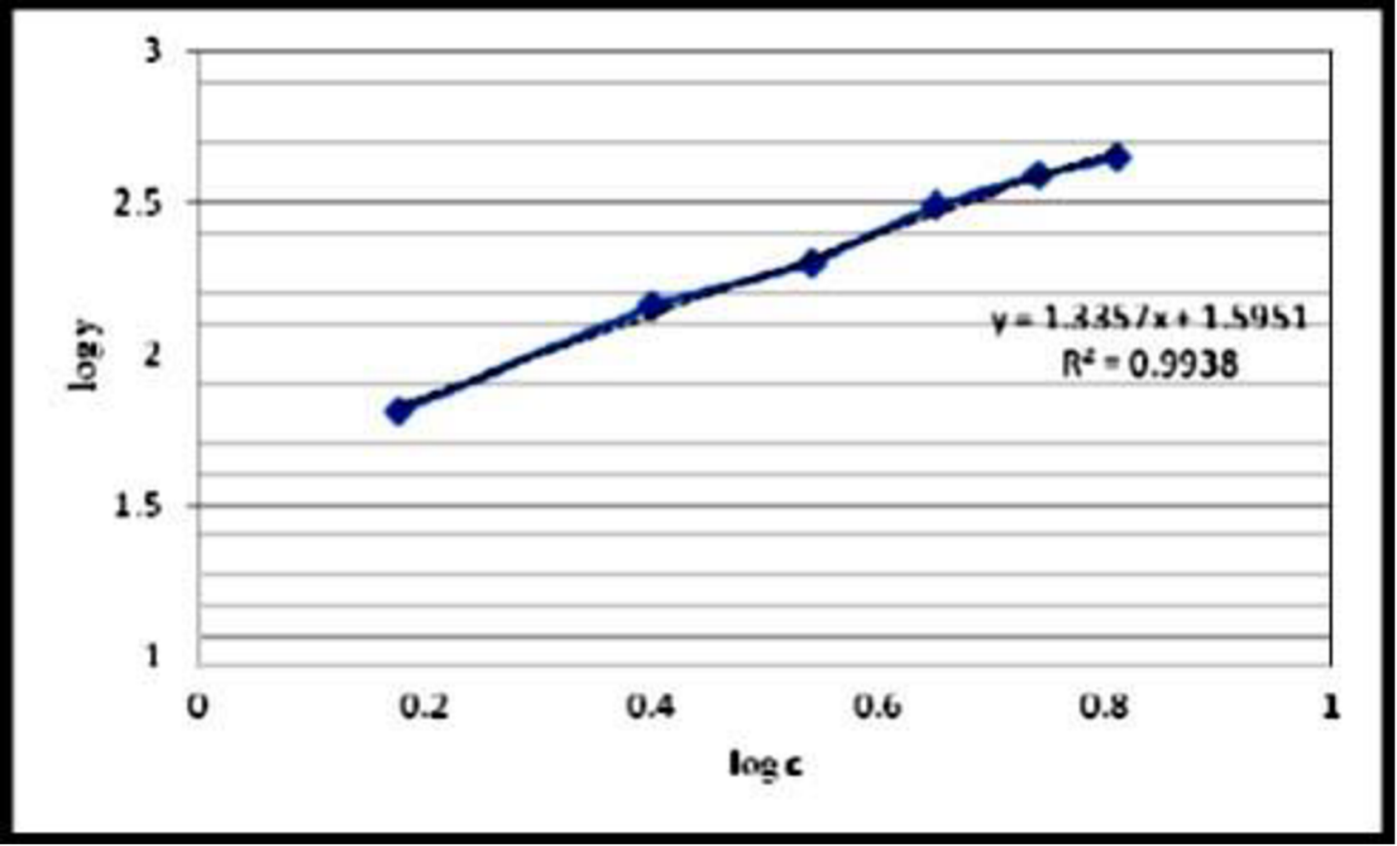
Catalytic activities of sample 6 in three concentrations. (a) 4.5 g. L ^-1^, (b) 9.5 g. L ^-1^, and (c) 18.5 g. L ^-1^.

In this study, we prepared different concentrations of sample by diluting it with n-hexane to investigate its effect on the sorption kinetics during the experiment. The catalytic activities of the obtained particles are evaluated by desulfurization of liquid fuels, and the adsorption amounts are changed by concentration of nanoparticles, as shown in Fig 11. Increasing of nanoparticles concentration from 4.5 g. L ^-1^ to 18.5 g. L ^-1^ was caused to increase the adsorption rate of sulfur contents of fuel. The adsorption rate of sulfur contents of fuel in various concentrations 4.5, 9.5, and 18.5 g. L ^-1^ of SrCr_x_Fe_12-x_O_19_ nanoparticles in solution was estimated about 39 (a), 50 (b), and 62% (c) for 30 min, respectively. This reveals that SrCr_x_Fe_12-x_O_19_ nanoparticles have the potential to be used as a new kind of semiconductor catalysts.

### Catalytic mechanism

Longmuir isotherm assumes monolayer adsorption onto a surface containing a finite number of adsorption. The linear form of Langmuir isotherm equation is given as:

$$\frac{Ce}{qe}=\frac{1}{Q0b}+\frac{1}{Q0}Ce$$

Where C*e* is the equilibrium concentration of the adsorbate (mg/l), q*e* is the amount of adsorbate adsorbed per unit mass of adsorbent (mg/g), Q_0_ and b are Langmuir constant.

### Freundlich isotherm

The isotherm was another form of the Langmuir approach for adsorption on a heterogeneous surface. the linear form of Freundlich equation is:

$$\log\;qe=\log\;kf+(\frac{1}{n})\;Ce$$

Where C*e* is the equilibrium concentration of the adsorbate (mg/l), q*e* is the amount of adsorbate adsorbed per unit mass of adsorbent (mg/g), K*f* and n are indicators of the adsorption capacity and adsorption intensity, respectively. The slope and the intercept of the linear frendlish equation are equal to 1/n and log K*f*, respectively. The percentages of sulfur removal calculated by the following equation:

$$Sulfur\;removal\;(\%)=\frac{S0-Se}{S0}\times 100$$

### Effect of temperature

**Fig 12:**
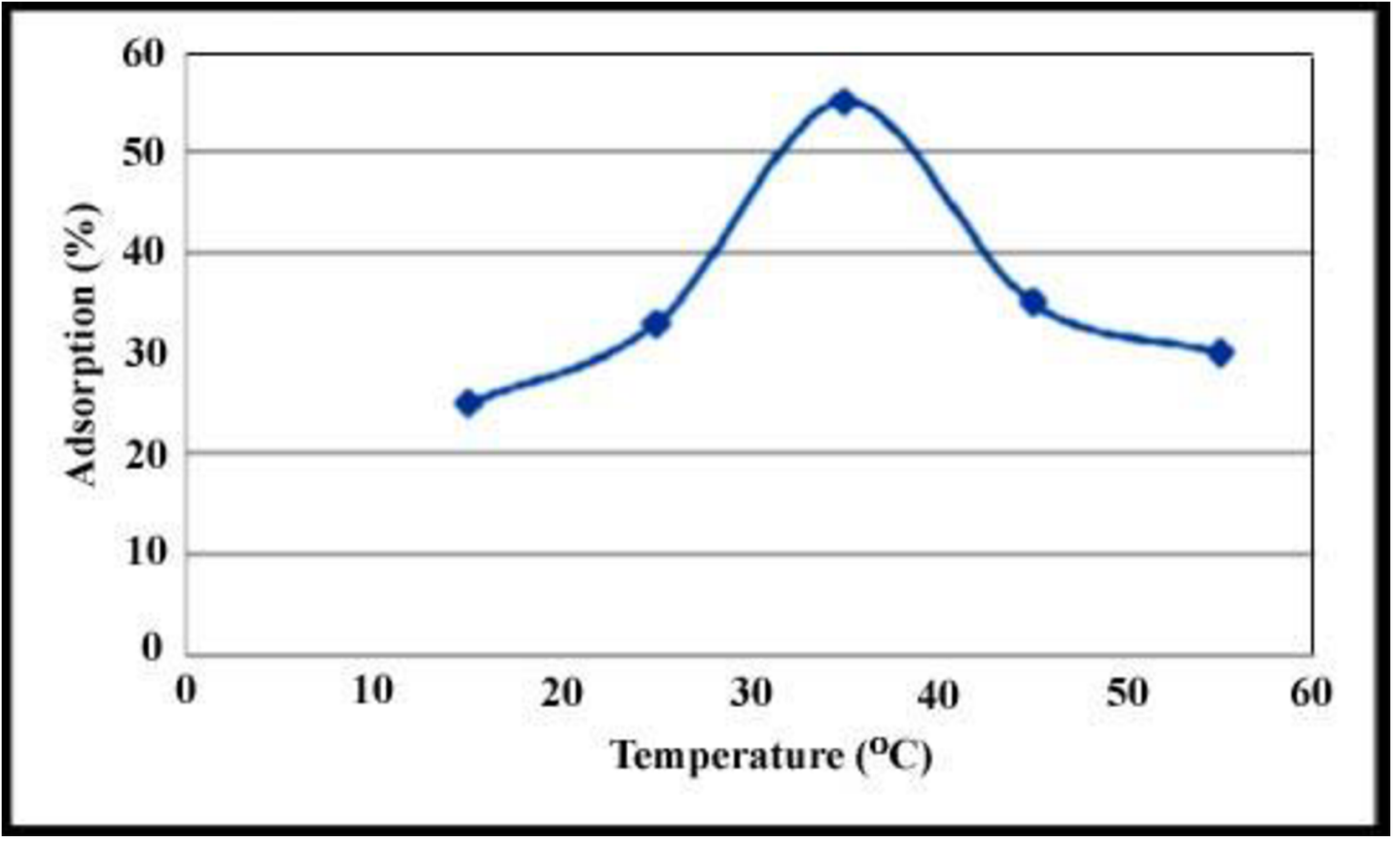
Effect of temperature on removal of total sulphur was studied at range 15–55°C.

Effect of temperature on removal of total sulphur was studied at range 15–55°C. The result shows that temperature has large effect on removal of total sulfur. Fig 12 shows that the residual sulfur concentration slightly increase when temperature was increase from 15 to 35°C. This indicates that there is activation energy for adsorption process. There is a decrease in sulfur adsorption whit temperature increasing from 35–55°C. It is possibly due to some sulfur desorption at higher temperature.

### Effect of contact time

**Fig 13:**
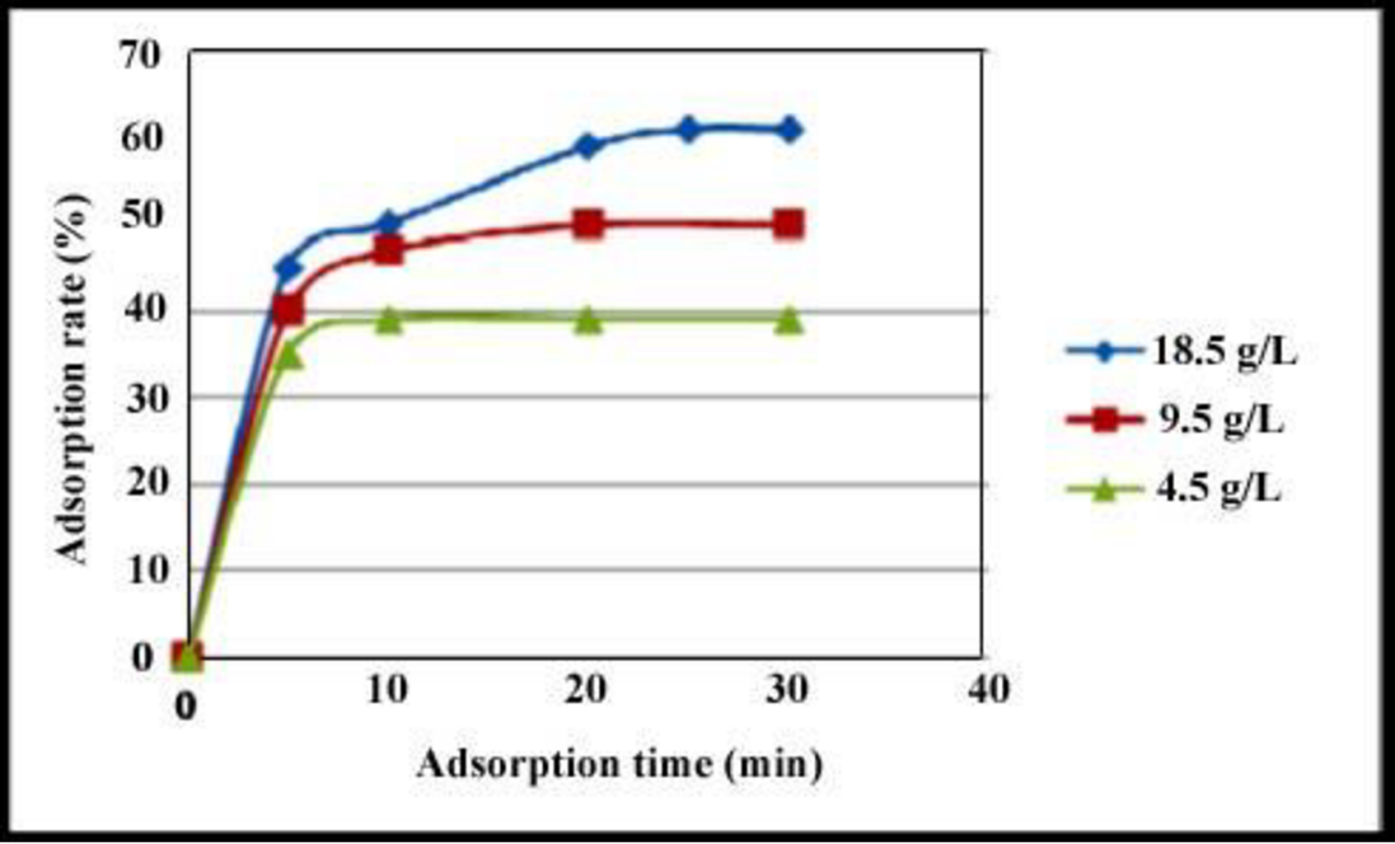
Effect of contact time on removal rate of sulfur at different concentrations of nanoparticles.

The effect of contact time on the rate of the removal of sulfur at different concentration of nanoparticle as shown in Fig 13 was investigated. The results indicates that the rate of sulphur removal was rapid in the beginning but it gradually decreased with time and at some point in time it reaches a constant value beyond which no more sulfur is further removed from the solution.at this point it reaches dynamic equilibrium and the amount of sulfur was absorbed indicated adsorption capacity.

## Conclusions

Chromium substituted strontium ferrites have been produced via a sol-gel auto-combustion technique by using various amino acids as fuel, reducing and capping agent. The effect of types of amino acid and chromium ion concentration on the morphological, magnetic and optical properties of products were studied. By considering SEM images, valine amino acid is chosen as desired amino acid for reducing process in auto-combustion reaction. XRD results are shown that when the concentration of Cr ^3+^ is ≥ 0.5, except than SrFe_12_O_19_ phase, Fe_2_O_3_ is formed. The saturation magnetization decreases with doping chromium to compare with SrFe_12_O_19_, due to the substitution of Cr ^3+^in the 12k and 2a sites of the lattice. Also, with doping of Cr ^3+^, coercivity is increasing due to increase in anisotropy field, which in turn increases of domain wall energy and morphology anisotropy. Investigating of UV-vis spectrum detected two absorption peaks around approximate 275 and 370 nm can be observed, which is similar to other reports. The present study shows that SrCr_x_Fe_12-x_O_19_ nanoparticle could be used as adsorbent for the desulfurization of liquid fuels. Increasing of nanoparticles concentration was caused to increase the adsorption rate of sulfur contents of fuel. The adsorption rate of sulfur contents of fuel in various concentrations 4.5, 9.5, and 18.5 g. L ^-1^ of SrCr_x_Fe_12-x_O_19_ nanoparticles in solution was estimated about 39, 50, and 62% for 30 min, respectively. The results of catalytic tests reveals that SrCr_x_Fe_12-x_O_19_ nanoparticles have the potential to be used as a new kind of semiconductor catalysts for the desulfurization of liquid fuels.
